# Automated Pavement Condition Index Assessment with Deep Learning and Image Analysis: An End-to-End Approach

**DOI:** 10.3390/s24072333

**Published:** 2024-04-06

**Authors:** Eldor Ibragimov, Yongsoo Kim, Jung Hee Lee, Junsang Cho, Jong-Jae Lee

**Affiliations:** 1 SISTech Co., Ltd., Seoul 05006, Republic of Korea; ibragimoveldor@gmail.com (E.I.); yskim4257@gmail.com (Y.K.); 2 Department of Artificial Intelligence, Ajou University, Suwon-si 16499, Republic of Korea; konide@ajou.ac.kr; 3 Korea Expressway Corporation Research Institute, Hwaseong-si 13550, Republic of Korea; junsangcho@ex.co.kr; 4 Department of Civil & Environmental Engineering, Sejong University, Seoul 05006, Republic of Korea

**Keywords:** pavement condition index, crack detection, deep learning, image processing, crack width estimation, pavements, crack segmentations, skeleton algorithm

## Abstract

The degradation of road pavements due to environmental factors is a pressing issue in infrastructure maintenance, necessitating precise identification of pavement distresses. The pavement condition index (PCI) serves as a critical metric for evaluating pavement conditions, essential for effective budget allocation and performance tracking. Traditional manual PCI assessment methods are limited by labor intensity, subjectivity, and susceptibility to human error. Addressing these challenges, this paper presents a novel, end-to-end automated method for PCI calculation, integrating deep learning and image processing technologies. The first stage employs a deep learning algorithm for accurate detection of pavement cracks, followed by the application of a segmentation-based skeleton algorithm in image processing to estimate crack width precisely. This integrated approach enhances the assessment process, providing a more comprehensive evaluation of pavement integrity. The validation results demonstrate a 95% accuracy in crack detection and 90% accuracy in crack width estimation. Leveraging these results, the automated PCI rating is achieved, aligned with standards, showcasing significant improvements in the efficiency and reliability of PCI evaluations. This method offers advancements in pavement maintenance strategies and potential applications in broader road infrastructure management.

## 1. Introduction

The vast network of roads plays a critical role in modern society, supporting essential activities from commerce to critical emergency services [1,2]. The economic and social stability of communities heavily relies on the robustness and reliability of these transportation infrastructures. Yet, the integrity of road networks is frequently compromised by deteriorating pavement conditions, leading to increased road accidents and substantial economic repercussions [3]. The degradation of pavements over time, due to environmental and operational stresses, necessitates a vigilant approach to their maintenance for the safety and efficiency of transportation systems.

Within the domain of road maintenance, the systematic assessment of pavement conditions is indispensable. Traditional pavement inspection methods, although historically effective, are marred by their subjective nature, labor intensity, and inherent risks, particularly in high-traffic environments. Technological advances have led to the adoption of sensor-equipped vehicles, offering a leap in the safety and efficiency of data collection. However, these methods often do not translate into standardized measures of pavement condition assessment—such as the pavement condition index (PCI)—which are essential for maintenance planning and economic allocation.

The focus of this study is to address the gap in the application of standardized and practical PCI evaluation methods. Although the current approaches have made progress in assessing the extent and density of pavement cracks, they often fall short of integrating these measurements into a standardized PCI framework recognized for infrastructure management and decision making. There is a clear need for a methodology, which not only enhances crack detection through advanced imaging techniques but also consolidates these data into a standardized PCI evaluation.

To this end, a novel automated method is proposed for evaluating pavement conditions, which employs a novel application of images captured by road inspection vehicles [4]. This method is predicated on a hybrid approach, which combines the precision of deep learning with the robustness of image processing techniques to detect cracks efficiently and accurately. This research advances the field by applying a comprehensive assessment framework, which aligns with real-world imaging conditions, thereby ensuring the practical relevance of the data collected.

The primary goal is to automate the calculation of the PCI, employing a wide array of pavement distress indicators to achieve a detailed and accurate portrayal of pavement conditions. This approach is crucial for informed decision making in road maintenance and infrastructure management. The method introduces a three-stage image-based process for PCI calculation, which begins with the detection of pavement cracks using the latest iteration of the “You Only Look Once” (YOLO) algorithm, YOLOv8. This is followed by the estimation of crack widths and culminates in the PCI calculation, demonstrating a level of robustness and adaptability superior to traditional methods. The study adheres to the AASHTO R 85-18 [5] and ASTM D6433-18 [6] standards, ensuring that the methodology is both consistent with industry norms and cost-effective.

The remainder of this paper is structured as follows. Section 2 reviews the related work, focusing on current deep-learning-based methods for pavement condition assessment and highlighting significant contributions and gaps in the field. Section 3 details the methodology of the proposed framework for automated pavement condition index evaluation, including data collection, crack detection algorithms, and the processes for width estimation and PCI calculation. Section 4 presents the experimental verification of the proposed method, illustrating the dataset preparation, processing, and results of crack detection and PCI calculation. Section 5, which encompasses the discussion and conclusions, assesses the implications of the automated approach and outlines future research directions in image-based pavement condition assessment.

## 2. Related Works

The assessment of pavement condition is a critical area in civil engineering, which has experienced substantial advancements in recent decades. Traditionally reliant on manual inspection, this field has increasingly adopted automated methods, enhancing accuracy and objectivity. This section reviews the literature on automated pavement condition assessment methods, emphasizing deep learning algorithms and image processing techniques.

Early studies in the automated detection of pavement distresses primarily focused on crack detection, employing image-based techniques, such as intensity thresholding and edge detection. Tang and Gu [7] demonstrated the use of intensity thresholding for crack identification, while Ayenu-Prah et al. [8] and Attoh-Okine et al. [9] highlighted edge detection’s utility in delineating crack boundaries. Further advancements were made with wavelet transforms (Chambon et al. [10]) and texture analysis (Hu and Zhao [11], Ojala et al. [12]), which refined crack detection and analysis. These foundational methods evolved into sophisticated machine-learning techniques for enhanced crack detection, as shown by Hoang and Nguyen [13]. 

To address the potential computational demands of complex crack detection algorithms, particularly in deep learning models, traditional image processing techniques offer the advantages of simplicity and lower resource requirements. While deep learning models can often achieve higher accuracy, image processing techniques still hold value due to their efficiency and accuracy in specific applications. This makes them particularly well-suited for scenarios where computational resources are constrained.

The expansion of data availability and computational power steered the focus toward deep learning methods. Deep learning approaches have been applied successfully in various tasks, including object detection, classification, and segmentation [14]. Notably, Mandal et al. [15] and Liu et al. [16] showcased deep-learning-based distress detection within pavement images. The field then progressed to employing specific one-stage detection networks, as demonstrated by Nie et al. [17] and Tran et al. [18]. Recent work on transformer-based networks [19] has also made substantial progress in crack detection, presenting an alternative to CNNs. These networks, leveraging self-attention mechanisms, improved general performance. Chen et al. [20] adopted a ViT approach for asphalt pavement image classification, designing a LeViT-based network architecture, which integrated convolution layers, an MLP-based pyramid-shaped transformer stage, multi-head self-attention blocks, and dual classifier heads. Hybrid solutions combining CNN and transformer-based methods have also emerged, with Luo et al. [21] and Zheng et al. [22] proposing a combination of YOLO and transformer networks for pavement damage detection.

The accurate detection and localization offered by deep learning methods led to the development of camera-based pavement condition assessment systems, focusing on quantifying pavement surface distresses and evaluating pavement conditions [23]. However, most research has concentrated on accurate distress recognition rather than direct pavement condition evaluation. Majidifard’s work [24] represents one of the first attempts to evaluate pavement condition using a condition rating system, employing a YOLO-based algorithm and U-Net for distress detection and segmentation with a dataset of 7237 Google Street images.

To balance the computational demands of complex crack detection algorithms with the need for detailed analysis, we employ a hybrid approach. Deep learning models are utilized for accurate crack detection, while image processing techniques offer a computationally efficient solution for segmentation. This approach leverages the strengths of both methods, making it well-suited for scenarios where computational resources may be limited.

Other researchers have proposed low-cost solutions using smartphones and customized camera setups. For instance, Roberts et al. [25] developed a deep-learning-based methodology for pavement condition monitoring using a smartphone mounted on a vehicle. This methodology was applied to a road network in Sicily, Italy, to identify different pavement distress types and their severities. Similarly, Mei et al. [26] employed a new camera system installed at the rear of a vehicle, focusing on crack segmentation for pavement inspection. Qureshi et al. [27] presented a comprehensive approach, which outputs pavement condition ratings on a scale from 0 to 100 using a dashboard-mounted camera system and incorporating image segmentation, data cleaning, resizing, cropping, and machine-learning-based classification.

Despite the advancements in automated pavement condition assessment, key limitations remain:

- Image quality: The necessity for high-resolution images is paramount due to the complex nature of crack patterns in pavement surfaces. Existing methods often fall short in capturing the requisite detail, leading to inaccuracies.
- Standardization: There is a notable lack of alignment with established standards, such as AASHTO or ASTM, in many current studies. This gap leads to inconsistencies and limits the comparability of assessment results across different systems.
- Subjectivity: Variability in expert interpretations of complex pavement conditions often results in subjective assessments, compromising the objectivity of evaluations.

The proposed end-to-end method addresses these limitations effectively. By employing high-resolution imaging, it captures detailed and complex crack patterns, enhancing detection accuracy. The system adheres to recognized industry standards, ensuring reliable and comparable assessment results. Furthermore, by automating the pavement condition index (PCI) calculation process, it significantly reduces the subjectivity associated with human expert assessments. This integration of advanced image processing and deep learning not only overcomes current challenges but also establishes a new benchmark for accuracy and standardization in pavement condition assessment, leading to more reliable infrastructure maintenance and management.

## 3. Methodology: Proposed Framework for Automated Pavement Condition Index Evaluation

**Figure 1:**
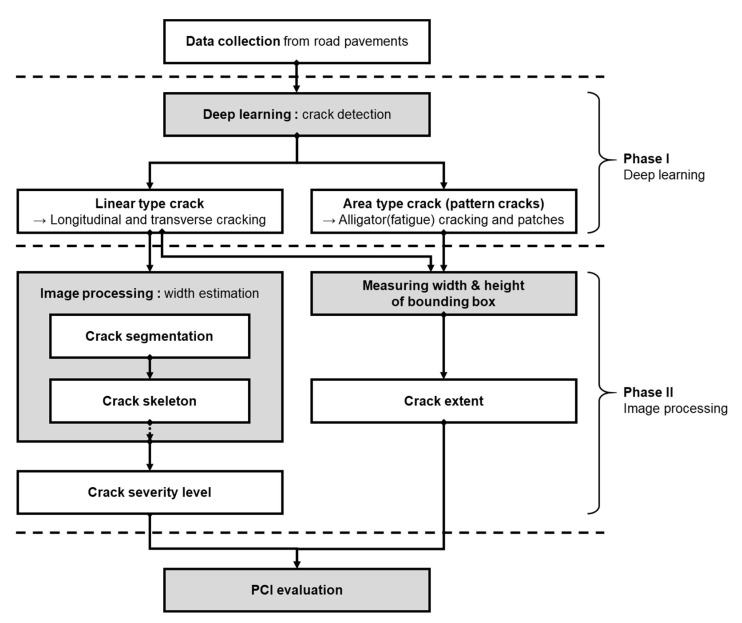
Flowchart of the proposed PCI calculation framework.

The objective of this work was the development of an automatic distress PCI calculation method and system, which uses information extracted from acquired pavement images. As shown in Figure 1, the operation of the proposed system consists of three stages: (1) crack detection, (2) width estimation, and (3) PCI calculation. As a main part of the system for the extraction of useful information (crack type and severity) from the acquired pavement images, two main modules are developed for crack detection and width calculation. For a given pavement image, the crack of interest is first detected, and its width is then evaluated. The detection module utilizes a YOLOv8 (see Section 3.1), which affords flexibility in terms of image size and state-of-the-art computer vision performance, especially for object detection. The crack width is then calculated to assess the severity level of road distress. For the width calculation, an image-processing-based algorithm is developed, which utilizes low-level image processing methods, such as segmentation and skeleton (see Section 3.2). The system then outputs critical information in the form of a bounding box, which defines the location of the crack in the image, the distress type (four types will be particularly defined in this paper), its extent (length and area), and its width. These details are fed into the automatic PCI calculator. The following is a detailed description of each stage of the proposed PCI calculation framework.

### 3.1. Crack Detection Using YOLOv8

**Figure 2:**
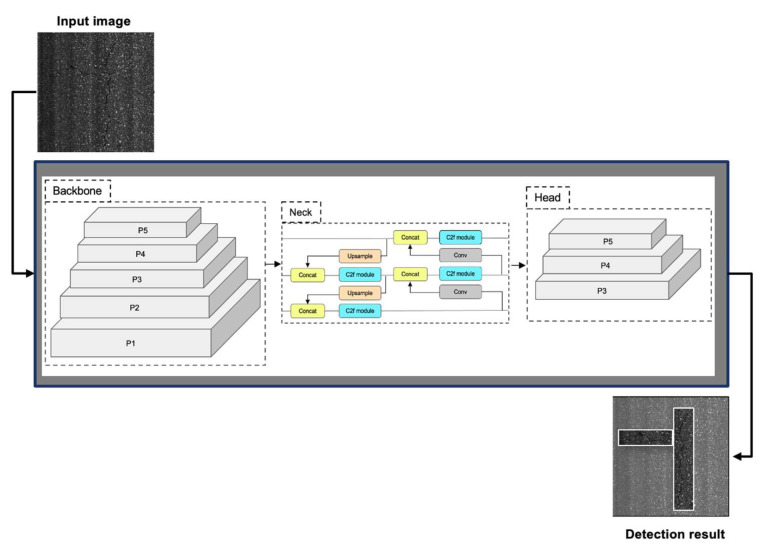
YOLOv8 architecture for pavement crack detection.

YOLOv8 is the latest version of the YOLO series [28,29,30]. It is a state-of-the-art object detection system, which is fast and accurate compared to its predecessors. It consists of four main components: input, backbone, neck, and output (see Figure 2).

The proposed method was developed in Python 3.11 using the PyTorch 2.1 deep learning framework, providing a flexible environment for pavement crack detection. The configuration of YOLOv8 from pre-processing to post-processing was meticulously adapted to meet the demands of pavement condition assessment. Details regarding the specific model configuration are discussed in Section 4.

The input component applies some pre-processing techniques to the input image to enhance the quality and diversity of the data. These techniques include mosaic data augmentation, which randomly crops and stitches four images together to form a new image, and adaptive anchor calculation, which adjusts the anchor boxes according to the distribution of ground truth boxes. Adaptive grayscale padding is applied, filling the padded areas with the image’s mean pixel value, to ensure consistency in the input data for subsequent processing steps.

The backbone component is the core of the network, which extracts features from different levels of the image. The backbone component uses a modified version of the C3 module, called C2f, which reduces the computational cost and enhances gradient flow. The C2f module incorporates the benefits of the efficient layer aggregation network (ELAN) structure in YOLOv7 [31], reducing one standard convolutional layer and making full use of the bottleneck module to enhance the gradient branch. The backbone component also uses a spatial attention module (SAM) to enhance the feature maps by assigning different weights to different regions based on their importance.

The neck component acts as an intermediary processing step, following the backbone network, which fuses features from different scales of the image. The neck component employs a feature pyramid network (FPN) [32] and a path aggregation network (PAN) [33] to fuse features from different levels of the network. This enables the model to capture both semantic and localization information and handle objects of varying sizes and shapes. FPN uses a top-down path to propagate high-level features to low-level features, and PAN uses a bottom-up path to propagate low-level features to high-level features. Both paths use adaptive feature fusion, which is a weighted sum of the features from the previous and the next levels. The neck component also adopts a spatial pyramid pooling fusion (SPPF) module to aggregate context information from different regions of the feature maps.

The head component is the part, which predicts the class and location of each object using decoupled heads, which separate the classification and regression tasks. The head component uses distribution focal loss (DFL) and CIoU loss, which are loss functions that aim to improve the performance of object detection. DFL is a variant of focal loss, which assigns different weights to different samples based on their difficulty. CIoU loss is a variant of IoU loss, which measures the similarity between the prediction box and the ground truth box. The head component also uses a task alignment metric, which combines the classification score and the IoU value, to select the best prediction boxes.

The output component is the part, which selects the best prediction boxes based on a task-aligned assigner, which assigns positive and negative samples based on a combination of classification and regression scores. The output component also uses some post-processing steps, such as non-maximum suppression (NMS) and soft-NMS, which eliminate duplicate detections and suppress low-confidence ones.

### 3.2. Width Calculation

**Figure 3:**
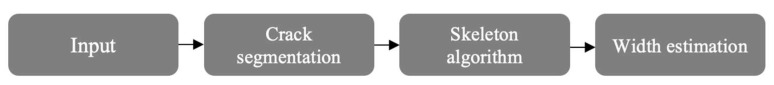
Steps in the width calculation process.

To calculate the PCI, crack widths are estimated in the second module of the proposed system. The crack width estimation algorithm employs the Sato Tubeness filter, implemented using the Python-based skimage [34] image processing library, to effectively isolate crack edges against noisy background textures. This is followed by skeletonization using an OpenCV algorithm [35]. The process involves crack segmentation followed by application of the skeletonization algorithm (see Figure 3 for a detailed workflow).

#### 3.2.1. Crack Segmentation Process

The crack segmentation process is initiated via application of the Sato Tubeness filter. This filter is applied due to its effectiveness in identifying linear patterns indicative of pavement cracks. The specifics of the filter’s operation and its combination with subsequent morphological operations, which are crucial for achieving precise segmentation, are discussed in order to facilitate accurate width calculation.

1. Sato Tubeness filter

The crack segmentation process is performed using the Sato Tubeness filter, a method tailored for 2D image analysis. It is particularly effective for delineating linear structures, such as pavement cracks, offering flexibility to accommodate varying crack widths. This adaptability is crucial for accurately segmenting different types of pavement cracks in the images.

Before computing the Hessian matrix *H* at each pixel over a range of scales σ, scales are chosen to match the expected variety in crack sizes. These scales are critical for capturing the full breadth of crack dimensions present in the pavement.

At scale *σ*, the Hessian matrix is defined as

(1) $$\;\;H\left(i,j,\sigma \right)=\left(\begin{array}{cc} L_{xx}\left(i,j,\sigma \right) & L_{xy}\left(i,j,\sigma \right)\\ L_{yx}\left(i,j,\sigma \right) & L_{yy}\left(i,j,\sigma \right) \end{array}\right)$$

In this matrix, $L_{xx},\;{\;L}_{xy}{,\;L}_{yx},\;$ and $L_{yy}$ represent the second-order partial derivatives of image intensity, providing key information on the curvature and orientation of structures within the image.

The eigenvalues $l_{1}$ and $l_{2}$ are derived from *H*, where a pronouncedly greater $l_{1}$ in comparison to $l_{2}$ is emblematic of a ridge structure/characteristic crack formation. The filter’s response at each pixel is given by

(2) $$\;\;\;\;\;\;\;\;\;\;\;\;\;\;\;\;\;\;\;\;\;\;\;\;\;\;\;\;\;\;\;\;\;\;\;R\left(i,j,\sigma \right)=\sigma^{2}\cdot \left(max\left(\lambda_{1}\left(i,j,\sigma \right),0\right)\right)\;\;$$

This response, R, is a measure of the likelihood that a pixel at location and scale σ belongs to a ridge-like structure. The multiplication by $\sigma^{2}$ serves as a scaling factor, enhancing the detection of larger features at larger scales.

The final segmentation result, $F\left(i,j\right)$, is obtained by selecting the maximum filter response across the scales:

(3) $$\;\;\;\;\;F\left(i,j\right)={max}_{sÎS\;}R(i,j,\sigma )$$

By selecting the maximum response, $F\left(i,j\right)$ ensures that the most prominent ridge-like feature at each pixel is captured, accurately representing the crack structures in the pavement image.

2. Morphological operations

Morphological operations are subsequently applied to the filtered image to refine the segmentation. These operations include dilation to connect fragmented cracks, erosion to reduce noise, and opening to smooth the crack paths, thereby enhancing the segmentation’s precision.

Following the application of the Sato Tubeness filter, the isolated noises and small holes within the crack structures are addressed. Morphological operations are applied to the binary image resulting from the Sato filter to improve the segmentation of the crack structures.

The process involves three steps using a binary image, denoted by *A*, and structural elements, denoted by *B*:

*1.*  Dilation is used to enhance the size of the cracks, aiding in bridging gaps within the crack regions. It is defined as

(4) $$\left.\;A⊕B=\left\{z|\left[{\left(\hat{B}\right)}_{z}\cap A\right)\right]\neq \emptyset \right\}$$

*2.*  Erosion serves to refine the crack paths by eliminating small noises. It is described by

(5) $$A\circ B=\left(A⊖B\right)⊕B$$

*3.*  Opening combines erosion and dilation to smooth the crack contours further and is represented as

(6) $$A⊖B=\left\{z|{\left(B\right)}_{z}\subseteq A\right\}$$

These steps, when applied in sequence, enhance the segmentation by cleaning up the noise and defining the cracks more clearly, which is crucial for accurate pavement condition assessment.

#### 3.2.2. The Skeleton Algorithm and Width Estimation

The skeleton method is mainly used to determine the topological properties of the crack, such as the length, direction, and width. The skeleton algorithm is employed [36] for width calculation using the parallel iterative thinning method [37]. A skeleton algorithm can be used to process binary segmented images by iteration through the image pixels to decrease the pixel width based on its direction, as much as down to one pixel. This made it particularly suitable for the proposed method. Furthermore, the shapes of the segmented cracks resembled a single linear line, and this made the shape extraction easier. The skeleton algorithm was successfully used to extract the skeletons of the binary segmented crack images. The average width of a crack was subsequently calculated by using the following equation:

(7) $$\;W=\frac{A}{B}$$

where *W* is the average width of the crack; *A* is the number of pixels in the crack; and *B* is the number of pixels in the skeleton of the crack.

### 3.3. PCI Calculation

In this section, the process of PCI evaluation is outlined. Initially, the criteria for assessing pavement condition are presented. This is followed by a brief overview of the standards, which inform the methodology. Finally, the section details the step-by-step process of PCI calculation, providing a clear and methodical approach to the evaluation method.

There are four different criteria for evaluating pavement condition: (1) the PCI; (2) surface characteristics, such as roughness, texture, and friction; (3) sub-surface characteristics; and (4) structural characteristics. This study focuses on the PCI, which indicates pavement surface distress. While the PCI does not encompass conditions such as roughness and structural capacity, it serves as a primary basis for determining the necessary maintenance or restoration work, including the assessment of the extent of pavement distress.

PCI evaluation primarily relies on three main criteria: (1) distress type, (2) severity, and (3) extent.

- Distress type: The identification of surface cracks and their types is crucial for pavement condition assessment. Different distress types are defined for PCI calculation based on their location and pattern. This study predominantly follows the AASHTO R 85-18 standard, which classifies distress into four types: (1) alligator pattern, (2) patching pattern, (3) longitudinal, and (4) transverse cracks.
- Severity level: As previously mentioned, there are four distress types under consideration. Longitudinal and transverse cracks are typically observed in the early stages of pavement deterioration. Detecting them in a timely manner allows for the application of minor restorative treatments, such as thin overlay and sealing. However, accurately measuring the severity level is crucial at this stage. Fortunately, this can often be achieved by measuring the crack width. Table 1 provides different severity levels based on crack width for longitudinal and transverse cracks. In the case of alligator and patching damage, their evaluation directly considers the urgency of restoration needs. Furthermore, for these distress types, there are limited viable restorative strategies, including costly pavement surface reconstruction. As discussed later in this study, a crack width calculation method is proposed for longitudinal and transverse cracks.
- Extent: The extent of pavement distress, particularly pattern cracks, such as alligator and patching damage, is evaluated based on its structural appearance on the pavement surface. This study thoroughly investigates the extent of deterioration through PCI calculations for these distress types.

**Table 1:** Severity levels of longitudinal and transverse cracks.

Severity Level	Crack Width (mm)
Low	<5
Medium	5–20
High	>20

There are established standards for collecting and analyzing pavement surface data, offering two primary benefits: ensuring data collection consistency and accurate reporting of pavement data. Transportation organizations have widely adopted standards, with the American Society for Testing and Materials (ASTM) standards being commonly applied. These standards encompass data collection, distress classification, and PCI evaluation. However, some agencies may develop their measurement methods based on unique historical conditions or combine standards with other protocols. Nevertheless, two main standards are frequently applied to pavement condition data, as discussed below.

- AASHTO Standard: The American Association of State Highway and Transportation Officials (AASHTO) standards are invaluable for organized distress information collection from images, covering aspects such as type, severity, and extent. In this work, AASHTO R85-18 is employed, which serves as the standard for quantifying cracks in asphalt pavement surfaces using automatically captured pavement images. Additionally, AASHTO PP68 [38] is used for collecting pavement surface images for distress detection. These standards enable detailed identification and rating of various pavement distress types.
- ASTM Standard: Numerous ASTM standards are widely employed in pavement assessment. Given the focus on PCI, ASTM D6433-18 is adopted: Standard Practice for Roads and Parking Lots Pavement Condition Index Surveys. This standard guides the collection of pavement condition data to determine the PCI value and its corresponding rating (please refer to Table 2). Overall, the proposed approach partially follows AASHTO R85-18 and ASTM D6433-18 for distress classification and PCI calculation, respectively.

**Table 2:** PCI rating.

PCI	Rating
86–100	Good
71–85	Satisfactory
56–70	Fair
41–55	Poor
26–40	Very Poor
11–25	Serious
0–10	Failed

**Figure 4:**
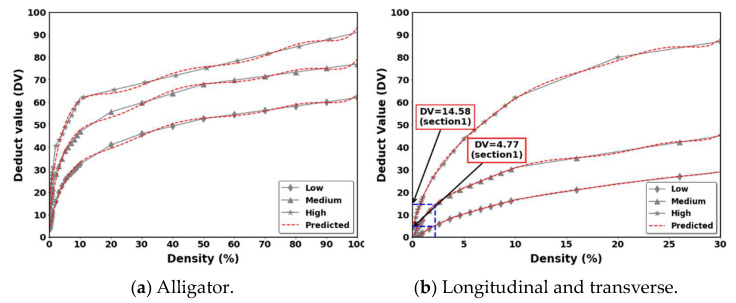

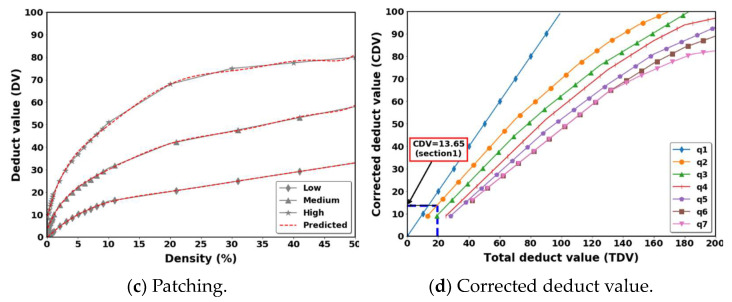
DVs and CDVs for the different distress types.

After the inspection of each pavement image using the detection and width estimation algorithm, the PCI was calculated. The calculation involved four main steps:

1. The *distress* type, severity level, and extent were determined for the sampled section. *Density* was then calculated using the following equation:
  (8) $$Density=\frac{Total\;distress\;amount}{section\;area}\times 100$$
2. Deduct value (DV) calculation: The DVs are calculated using deduction value curves provided in the ASTM D6433-18 standard. A higher deduct value indicates greater deterioration of the pavement structure. The deduct value is evaluated based on the distress type, severity, and density of the pavement. Because the present work was fully automatic, all the deduct values were digitized and fitted using a polynomial regression model.
3. Calculation of total DV (TDV): The TVD is calculated by summing the deduct values for each sampled unit. Every individual deduct value is determined by first classifying the density or extent of a distress according to its severity level (with q1 representing the lowest severity and q7 representing the highest). The appropriate deduct value curve in ASTM D6433 is then used to obtain the corresponding deduct value. The corrected deduct value (CDV) for the sample unit can then be determined from the curve shown in Figure 4. This process considers the severity levels determined by the q1–q7 classification, ensuring that the most severe distresses have the greatest impact on the final PCI.
4. PCI value: The PCI is determined by subtracting the CDV from the perfect score of 100.

## 4. Experimental Verification of the Proposed Method

This section encompasses a comprehensive description of the dataset preparation and processing for training the proposed model. It also presents the implementation details, evaluation metrics, detection experiments with both quantitative and qualitative results, as well as reporting the speed of the proposed method. Additionally, the width calculation is discussed, followed by PCI calculation for actual pavement images using the results of the crack detection and width calculation.

### 4.1. Dataset and Settings

The experiments were conducted in Seoul, South Korea, utilizing a specialized truck equipped with a camera designed to capture images of the pavement surface. This system operates at vehicle speeds up to 100 km/h with a sampling frequency ranging from 5600 Hz to a maximum of 11,500 Hz, ensuring high-resolution data capture. The image resolution is 3730 × 10,000 pixels. Images covering standard lane widths of 3–3.5 m and lengths of 10–10.5 m were selected to facilitate precise PCI calculations in alignment with established standards. The data extracted from the images included information regarding the distress type, such as longitudinal, transverse, alligator cracks, or patching. Due to the exceptionally high resolution of the images, each image was divided into 10 equal parts, each measuring 1865 × 2000 pixels. This not only eliminated small cracks but also reduced the memory footprint. In total, 3600 images were obtained from this process. Among these, 2600 images were allocated for training, 600 for validation, and 400 for testing purposes. Manual annotation of the acquired data was carried out using the LabelImg annotation tool [39]. The organization of annotations and data folders followed the structure of the popular Pascal VOC dataset [40], with folders created in the following order: (1) [JPEGImages], (2) [Annotations], and (3) [ImageSets] for training, validation, and testing, respectively.

In order to train the YOLOv8 model for crack detection, a transfer learning approach was employed, utilizing a pre-trained model [41]. This approach facilitated faster training with improved convergence, given that the dataset was relatively small in comparison to existing datasets comprising millions of images. Through this approach, the crack features were effectively extracted.

Hyperparameters were configured with a learning rate of 0.001, a weight decay of 0.0005, a total of 100 epochs, and utilization of an Adam optimizer [42].

### 4.2. Crack Detection Results

**Figure 5:**
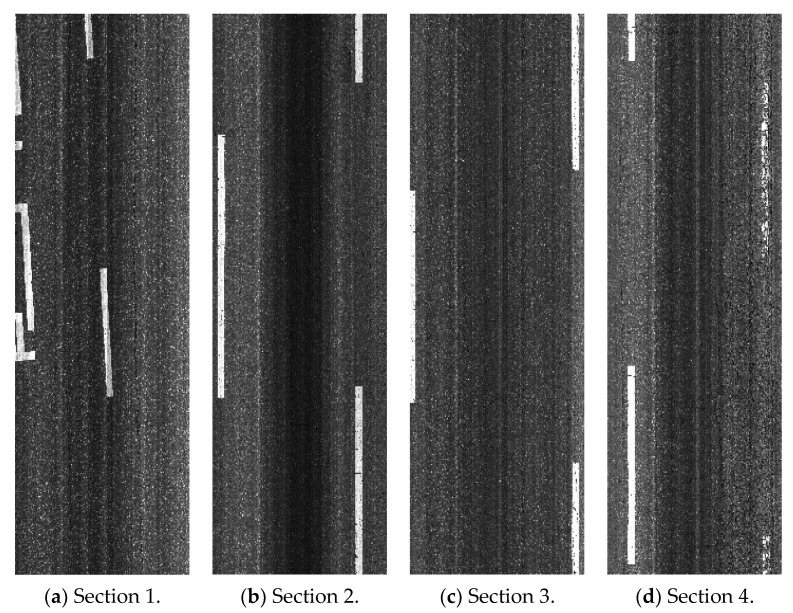
Sample pavement surface images.

Four typical pavement images (shown in Figure 5) were used for the performance estimation of the proposed method. Here, the images include different distress types (e.g., patching, longitudinal, transverse, and alligator cracks). The columns from left to right correspond to the pavement sections, including different crack densities. (Section 1 (low density), Section 2 (low density), Section 3 (high density), Section 4 (high density)).

The performance of the detection model was examined in this work based on two common evaluation metrics: (1) pixel accuracy and (2) mean intersection union (*IU*). For both evaluation metrics, the true positive (*TP*) is the correct prediction of pixels with correct ground truth pixels; the true negative (*TN*) is the correct prediction of pixels with non-ground-truth pixels; the false positive (*FP*) is the incorrect prediction of pixels with correct ground truth pixels; and the false negative (*FN*) is the incorrect prediction of pixels with incorrect ground truth pixels.

- *Pixel accuracy:* This is a simple metric mainly based on the comparison of each pixel with the ground truth and detection result images. In this case, the overall pixel values are calculated by considering all the pixels to be of the same type.

(9) $$pixelaccuracy=\frac{TP+TN}{TP+TN+FP+FN}$$

- *Mean IU (mean IU)*: The *mean IU* is calculated for each distress type, and then, the average is taken across all distress types.

(10) $$mean\;IU=\frac{1}{N}(\frac{TP+TN}{TP+FP+FN})$$

where *N* is the number of distress types in the evaluation data.

**Figure 6:**
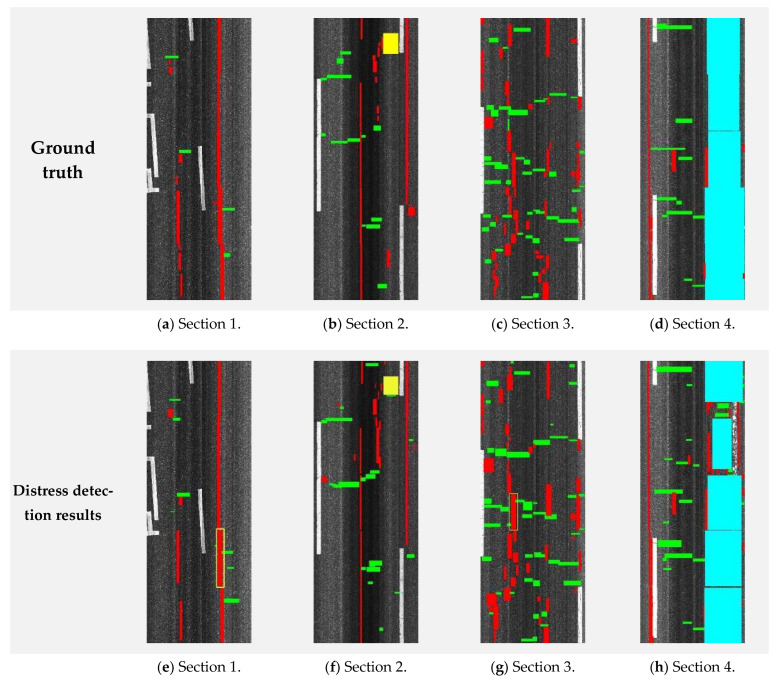
Comparative visualization of ground truth and distress detection results across four pavement sections.

The crack detection results are shown in Figure 6 and Table 3. In the figure, the top and bottom rows, respectively, correspond to the ground truths of the original images and the present results. The columns from left to right correspond to the pavement sections (Section 1 (low density), Section 2 (low density), Section 3 (high density), Section 4 (high density)). The different colors indicate different distress types (i.e., red: longitudinal, green: transverse, yellow: patching, blue: alligator). The crack detection performance was evaluated quantitatively and qualitatively.

- Qualitative results: Qualitative results obtained using the proposed method for an image with a size of 3730 × 10,000 pixels are shown in Figure 6. For better visualization, the bounding boxes of the cracks are colored. Note that the proposed method performs well on these images as well, showing the applicability of the proposed method.
- Quantitative results: Table 3 gives the results for the images in Figure 6. The average pixel accuracy for the data was 95%, while the mean IU was 66%.
- Processing speed: A further examination of the speed of the proposed crack detection method was conducted based on a single image. An image with a size of 3730 × 10,000 pixels was found to require approximately 2 s for the detection process. This shows that the method is practical.

### 4.3. Results for Width Calculation and Crack Segmentation

This section details the methodology employed for calculating crack widths from pavement images, which forms a key part of the crack detection process developed in this study. A set of 300 images, specifically selected for their inclusion of transverse and longitudinal cracks, were analyzed. Ground truth data, essential for validating the detection method’s performance, were meticulously gathered through manual visual inspection of the pavement. These data underwent a labeling process in two distinct stages: the initial binary segmentation of cracks followed by the measurement of their widths from the segmented results. The camera used for capturing the pavement images was calibrated such that one pixel equated to a length of one millimeter, ensuring that the segmentation and subsequent width measurements could be precisely correlated with real-world dimensions.

The effectiveness of the crack width calculation method is highly dependent on the binary segmentation’s ability to distinguish cracks within the images. The developed algorithm is specifically designed to identify cracked regions, which are depicted in Figure 6. The experimental data affirm the method’s robust performance across a range of pavement conditions, including scenarios with noisy textures and low contrast between the cracks and the pavement background.

The method was determined to have an average accuracy of 95%. After segmentation, the width calculation method was applied, which utilized the skeleton algorithm. The skeleton information and segmentation output were used to estimate the width using Equation (7). The previously generated ground truth data were used to examine the performance of this method, which was found to have a width estimation accuracy of 90%. To calculate the PCI, which was the actual interest of this study, the width calculation results were sorted out by severity levels using Table 1.

### 4.4. PCI Calculation and Analysis

Utilizing the results from the automatic detection and width calculation algorithm, the PCI for each pavement section was computed following the ASTM D6433-18 standards. Each section was represented by high-resolution images, with the dimensions of captured road pavement sections ranging from 3.5 to 3.7 m in width and 10 m in length, as illustrated in Figure 5.

The calculation of PCI was conducted as per the methodology detailed in Section 3.3, which outlined the necessary steps and equations for assessing pavement condition. The process included sorting crack information by type, extent, and severity, determining deduct values, applying a polynomial regression model to the DVs to ascertain the best fit, and calculating the corrected deduct values (CDVs). The final PCI was then computed by subtracting the CDV from a perfect score of 100.

**Figure 7:**
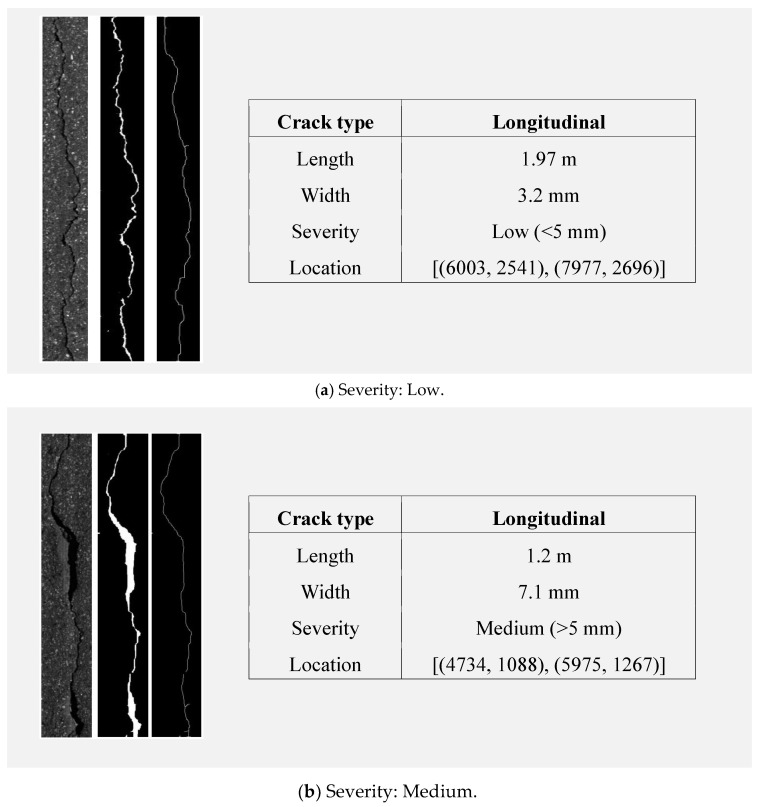
Results of the crack segmentation, skeletonization, and quantification.

The collected data, illustrated in Figure 7, present a sequence of images outlining our crack analysis workflow, from the original cropped image to binary segmentation and final skeletonization, with a table summarizing the crack’s type, dimensions, severity, and location. Table 4 demonstrates varied pavement conditions across the sampled sections. Sections 1 and 2, with lower crack densities, exhibited “good” and “satisfactory” conditions, respectively. Interestingly, Section 3, despite being categorized as high density, also displayed “satisfactory” conditions due to the predominance of low-severity cracks. In contrast, Section 4 was rated as “very poor” due to extensive alligator cracking, emphasizing the critical nature of such distress in the PCI assessment.

By implementing the steps outlined earlier, this section presents the practical application and results of the PCI calculation, reinforcing the effectiveness of the automated system in providing accurate and reliable pavement condition assessments.

## 5. Discussion and Conclusions

The research presents a significant leap in the domain of civil infrastructure analysis through the development of an automated approach for evaluating the pavement condition index (PCI). Applied in Seoul City, South Korea, this method, adhering to AASHTO R85-18 and ASTM D6433 standards, represents a breakthrough in combining high-resolution imaging with advanced image processing techniques. Despite its effectiveness, it is important to note that the current data acquisition system is relatively costly. The methodology, primarily focused on major pavement distress types, demonstrated accuracy in classifying crack types and severity levels, as well as superior processing speeds compared to traditional methods.

The use of high-resolution images, while crucial for precision in pavement condition assessments, contributes to the higher cost of the current system. This aspect is particularly vital in real-world scenarios for accurate detection of pavement distresses. This method, with its current focus on major distress types, addresses the most frequently encountered issues in urban settings. However, we recognize the potential for future enhancements, including the possibility of transitioning to a more cost-effective, customized line-scan-based camera system. This future development could offer a more economically feasible solution while maintaining, or even improving, the accuracy and efficiency of the assessments.

As the field of deep learning continues to evolve and demonstrate robustness, replacing traditional image processing techniques with a fully deep-learning-based approach seems inevitable. This transition is expected to not only refine the robustness and efficiency of the PCI assessment process but also potentially reduce costs associated with data acquisition.

The deliberate focus on commonly observed pavement distresses, while strategic in maximizing practical relevance, is complemented by the adaptability of the methodology. Future research will expand this focus to include a broader range of pavement distress types and explore the integration of deep learning techniques, which will open new avenues for research and application. This evolution promises significant contributions to the efficiency and effectiveness of infrastructure maintenance and management strategies.

The implications of the study for pavement maintenance and infrastructure management are considerable. By automating the PCI calculation process and potentially shifting to a more cost-effective data acquisition system in the future, the proposed method stands to significantly reduce both the time and costs associated with manual inspections. The anticipated integration of deep learning techniques is poised to advance this technology further, suggesting a new benchmark in the field of infrastructure assessment.

In summary, this study not only addresses the current challenges in automated pavement condition assessment but also lays a solid foundation for future technological integration and expansion. The successful application of the proposed method in Seoul City underscores its efficacy in producing robust, accurate, and efficient evaluations of pavement conditions. Looking ahead, the expansion of distress types analyzed and the integration of emerging deep learning techniques, coupled with a potential shift to a more cost-effective data acquisition system, will significantly contribute to the efficiency and effectiveness of infrastructure maintenance and management. The contributions thus provide a promising direction for future innovations in pavement condition assessment, highlighting the potential for a fully automated, intelligent pavement evaluation system.

## Figures and Tables

**Table 3:** The detection results.

Image Section	Pixel Accuracy (%)	Mean IU (%)
Section 1	98.30	70.67
Section 2	96.48	71.15
Section 3	93.07	67.62
Section 4	91.69	54.67
Average	95%	66%

**Table 4:** Overall results of PCI evaluation.

Section No.	Distress Type	Severity	Length and Area	Density	DV	TDV	q	CDV	PCI	Pavement Condition
Section 1	Long. and Trans.	Low	75.56	2.04	4.77	19.35	2	13.65	86.35	Good
		Medium	81.48	2.2	14.58					
Section 2	Long. and Trans.	Low	91.26	2.47	5.76	34.99	3	20.28	79.72	Satisfactory
		Medium	133.64	3.61	18.87					
	Patching	Medium	0.47	1.26	10.36					
Section 3	Long. and Trans.	Low	191.75	5.18	10.52	27.13	2	19.37	80.63	Satisfactory
		Medium	102.93	2.78	16.61					
Section 4	Long. and Trans.	Low	133.64	3.61	18.87	104.73	3	65.23	34.77	Very Poor
		Medium	143.13	3.87	19.47					
	Alligator	High	10.76	29.11	73.67					

## Data Availability

This study did not report any specific data.
